# P-1819. Point Prevalence Study Is A Useful Tool to Evaluate Effectiveness of Antimicrobial Stewardship Program Strategies in Surgical Departments at Singapore General Hospital

**DOI:** 10.1093/ofid/ofae631.1982

**Published:** 2025-01-29

**Authors:** Peijun Yvonne Zhou, Jia Le Lim, YiBo Wang, Jun Jie Tan, Nathalie Grace Sy Chua, Kai Chee Hung, Winnie Lee, Lai Wei Lee, Shena Yun Chun Lim, Yi Xin Liew, Li Wen Loo, Narendran Koomanan, Boon San Teoh, Daphne Yah Chieh Yii, Li Xuan Trevina Lee, Siew Yee Thien, Benjamin Pei Zhi Cherng, Maciej Piotr Chlebicki, Cherie Si Le Gan, Lay Hoon Andrea Kwa, Shimin Jasmine Chung

**Affiliations:** Singapore General Hospital, Singapore; Singapore General Hospital, Singapore; Singapore General Hospital, Singapore; Singapore General Hospital, Singapore; Singapore General Hospital, Singapore; Singapore General Hospital, Singapore; Singapore General Hospital, Singapore; Singapore General Hospital, Singapore; Singapore General Hospital, Singapore; Singapore General Hospital, Singapore; Singapore General Hospital, Singapore; Singapore General Hospital, Singapore; Singapore General Hospital, Singapore; Singapore General Hospital, Singapore; Singapore General Hospital, Singapore; Singapore General Hospital, Singapore; Singapore General Hospital, Singapore; Singapore General Hospital, Singapore; Singapore General Hospital, Singapore; Singapore General Hospital, Singapore; Singapore General Hospital, Singapore

## Abstract

**Background:**

In 2021, point prevalence survey (PPS) showed 51.2% inpatients at Singapore General Hospital (SGH) was prescribed ≥ 1 antibiotic, higher than many developed countries. Notably, % surgical inpatients on antibiotics were high at 59.7%. Among patients who received surgical prophylaxis (SP), 63.7% had SP > 24 hours. In addition to existing antibiotic stewardship program (ASP) strategies (e.g. prospective audit feedback, computerized clinical decision support system), ASP introduced a series of new strategies from 2021, involving digital solutions, handshake stewardship and project collaborations with surgeons to improve antibiotic prescribing [Figure 1].

**Figure 1.**
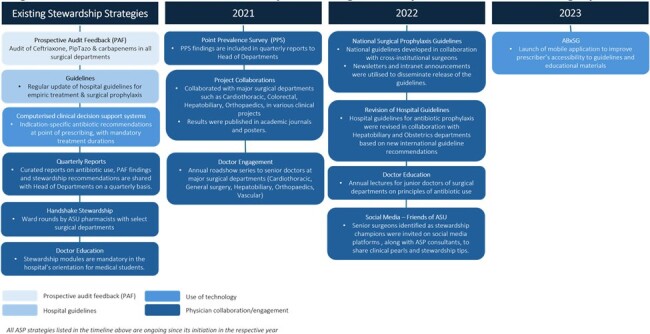
Antibiotic stewardship strategies implemented at Singapore General Hospital

**Methods:**

PPS was conducted annually using standardized Global-PPS protocol from 2021 to 2024 among surgical inpatients. The % of inpatients on antibiotics, antibiotic related information and quality indicators were collected. In addition, presence of surgical site infections (SSI), as defined by Centers for Disease Control and Prevention, was compared in patients prescribed ≤ 24 hours and > 24 hours of SP.

**Figure 2.**
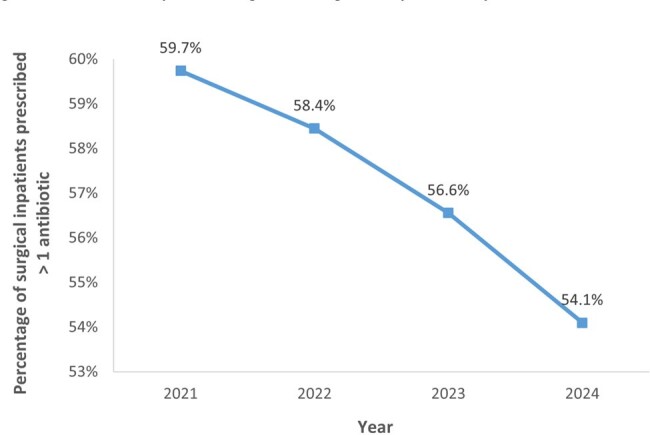
Trend of percentage of surgical inpatients prescribed > 1 antibiotic from 2021 to 2024

**Results:**

The % surgical inpatients prescribed with antibiotics steadily decreased from 59.7% (2021) to 54.1% (2024) [Figure 2]; an average of ∼1.9% reduction/year (translating to ∼3,500 antibiotic-free days/year). Notably, PPS showed improvement in documenting antibiotic indication, stop/review date and compliance to hospital antibiotic guidelines over time, suggesting that doctors were more cognizant about appropriate antibiotic prescribing [Figure 3].

In patients prescribed with SP, % patients with SP > 24 hours decreased from 63.7% (2021) to 52.2% (2023) [Figure 4]. There was no difference in SSI between patients who received ≤ 24 hours and > 24 hours of SAP (2/193, 1.0% vs 8/247, 3.2%, *p*=0.2).

**Figure 3.**
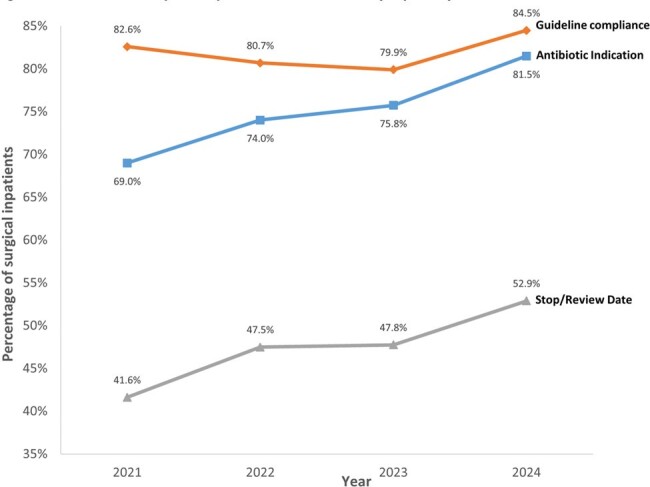
Trend of point prevalence survey quality indicators from 2021 to 2024

**Conclusion:**

PPS is a useful tool to evaluate the effectiveness of ASP strategies on antibiotic use. With the introduction of new digital solutions and reinforcing handshake stewardship via greater physician engagement and collaboration, % of surgical inpatients on antibiotics has reduced steadily over the years. The % patients prescribed > 24 hours of SP has also reduced. ASP must remain adaptable in integrating innovative initiatives to educate and engage prescribers to further improve antibiotic prescribing.

**Figure 4.**
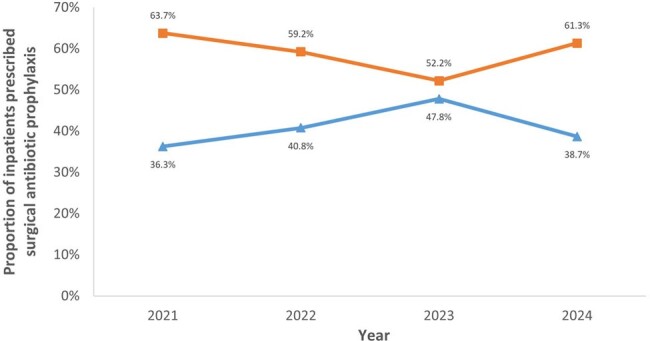
Trend of the duration of surgical prophylaxis from 2021 to 2024

**Disclosures:**

**All Authors**: No reported disclosures

